# Comparison of Reintervention Outcomes After Endoscopic Ultrasound-Guided Hepaticogastrostomy According to the Type of Initial Stent

**DOI:** 10.3390/jcm15218711

**Published:** 2026-11-08

**Authors:** Yukari Suzuki, Takashi Sasaki, Tsuyoshi Takeda, Masashi Miura, Masanori Nagasawa, Toyokazu Saito, Yuri Maegawa, Takafumi Mie, Takeshi Okamoto, Masato Ozaka, Naoki Sasahira

**Affiliations:** Department of Hepato-Biliary-Pancreatic Medicine, Cancer Institute Hospital, Japanese Foundation for Cancer Research, Tokyo 135-8550, Japan; yukari.suzuki@jfcr.or.jp (Y.S.);

**Keywords:** endoscopic ultrasound-guided hepaticogastrostomy, recurrent biliary obstruction, reintervention, plastic stent, self-expandable metallic stent

## Abstract

**Background/Objectives**: The impact of initial stent type on endoscopic reintervention after endoscopic ultrasound-guided hepaticogastrostomy (EUS-HGS) remains unclear. **Methods:** We retrospectively evaluated patients with malignant biliary obstruction who developed recurrent biliary obstruction (RBO) after EUS-HGS and underwent reintervention through the endosonographically created route (ESCR). Outcomes were compared according to initial stent type (plastic stent [PS] or self-expandable metallic stent [SEMS]). The association between initial stent type and time to RBO (TRBO) after definitive biliary drainage was assessed using Cox regression and Fine–Gray regression. **Results:** Of 170 patients who underwent EUS-HGS, 69 were included (PS, *n* = 23; SEMS, *n* = 46). The initial reintervention procedure was technically successful in 100% and 95.7% of patients, respectively (*p* = 0.55). Definitive biliary drainage was technically successful in all 69 patients, while clinical success was achieved in 62 (89.9%). No procedure-related adverse events were observed. Among patients with clinical success, median TRBO was 160 and 140 days in the PS and SEMS groups, respectively. Initial SEMS placement was not significantly associated with TRBO (adjusted hazard ratio, 1.32; 95% confidence interval [CI], 0.55–3.13) or the cumulative incidence of RBO (adjusted subdistribution hazard ratio, 1.67; 95% CI, 0.70–3.97). **Conclusions**: Endoscopic reintervention through the ESCR was technically feasible after both initial PS and SEMS placement. Initial stent type was not significantly associated with subsequent RBO, although the findings should be interpreted cautiously because of potential temporal confounding and the limited sample size.

## 1. Introduction

Endoscopic ultrasound-guided biliary drainage (EUS-BD) has become an important alternative to endoscopic retrograde cholangiopancreatography (ERCP) for malignant biliary obstruction (MBO), particularly when ERCP is unsuccessful or technically infeasible [1,2,3]. EUS-guided hepaticogastrostomy (EUS-HGS) is an effective biliary drainage option for patients with surgically altered anatomy, duodenal obstruction, or hilar biliary obstruction. For EUS-HGS, covered self-expandable metallic stents (SEMS) are generally recommended because of their longer stent patency and reduced risk of bile leakage [4,5]. Among these, partially covered SEMS (PCSEMS) are generally preferred over fully covered SEMS because of concerns regarding stent migration [6]. However, the optimal stent type for initial EUS-HGS remains unclear.

In EUS-HGS, SEMS are generally deployed with a sufficient intragastric length to prevent stent migration. However, a long intragastric portion can make subsequent endoscopic reintervention technically challenging. Previous studies have reported the feasibility of reintervention after EUS-HGS using covered SEMS, although reintervention may require access through the stent lumen or mesh openings at the gastric end [7,8,9]. In contrast, plastic stents (PS) have potential disadvantages, including bile leakage and shorter stent patency, but offer advantages in terms of stent placement, removal, and exchange. A mature endosonographically created route (ESCR) may develop after PS placement, permitting subsequent endoscopic reintervention through the ESCR [10,11].

With advances in systemic therapy [12,13,14,15], an increasing number of patients with MBO may require repeated biliary interventions during their disease course [16]. The initial stent type may therefore influence not only primary stent patency but also subsequent reintervention. Although SEMS generally provide longer primary patency than PS, their potential impact on subsequent reintervention remains unclear [17]. Few studies have examined whether the type of stent used for initial EUS-HGS affects reintervention outcomes and subsequent stent patency. Therefore, this study aimed to evaluate the association of initial stent type with reintervention outcomes and time to recurrent biliary obstruction (TRBO) after definitive biliary drainage.

## 2. Materials and Methods

### 2.1. Patients

We conducted a retrospective analysis of consecutive patients who underwent EUS-HGS for MBO between July 2016 and January 2026. Among these patients, those who subsequently developed RBO and underwent endoscopic reintervention through the ESCR were included. No patients with RBO underwent reintervention via ERCP or percutaneous transhepatic biliary drainage (PTBD).

Follow-up continued until death from any cause, loss to follow-up, or the data cutoff date (31 July 2026). Ethical approval for this study was obtained from the Institutional Review Board (IRB) of our institution (IRB number: 2023-GB-077), and was conducted in accordance with the principles of the Declaration of Helsinki. The requirement for informed consent was waived because of the retrospective study design.

### 2.2. Data Collection

We collected data on age, sex, Eastern Cooperative Oncology Group (ECOG) performance status, laboratory findings, disease characteristics, clinical outcomes, and adverse events (AEs) from electronic medical records. AEs were assessed from the initial reintervention or definitive biliary drainage, as applicable, until RBO, death, loss to follow-up, or the data cutoff date, whichever occurred first.

### 2.3. Endoscopic Procedures

EUS-HGS was performed using a convex echoendoscope (GF-UCT260; Olympus Medical, Tokyo, Japan, or EG-740UT; Fujifilm Medical, Tokyo, Japan). The SEMS used were the Niti-S Spring Stopper and Niti-S S-type (Taewoong Medical, Seoul, Korea), and the EGIS Biliary Stent (S&G Biotech, Yongin, Korea), all of which were PCSEMS. The PS used was the Through & Pass TYPE IT (Gadelius Medical, Tokyo, Japan). SEMS were predominantly used until December 2023, whereas PS became the preferred stent type from January 2024 onward. However, SEMS were preferentially selected for patients with ascites along the puncture route, even after January 2024. The numbers of patients receiving PS and SEMS were 4 and 42 between July 2016–December 2023, and 19 and 4 between January 2024–January 2026, respectively. The final choice of stent type was left to the discretion of the endoscopist.

Reintervention for RBO was performed using a duodenoscope (TJF-260V or TJF-Q290V; Olympus Medical, Tokyo, Japan, or ED-580T or ED-840T; Fujifilm Medical, Tokyo, Japan). In patients with an initial PS, the stent was exchanged for either a PS (Through & Pass, or Through & Pass TYPE IT; Gadelius Medical, Tokyo, Japan) or SEMS (Niti-S Spring Stopper; Taewoong Medical, Seoul, Korea, or HANAROSTENT Biliary Multi-Hole Benefit; M.I. Tech, Seoul, Korea). In patients with an initial SEMS who developed RBO due to tissue hyperplasia through the uncovered portion, an additional SEMS (HANAROSTENT; Boston Scientific, Marlborough, MA, USA, or Niti-S Large Cell D-type; Taewoong Medical, Seoul, Korea) or a PS (Through & Pass, Through & Pass Double Pigtail, Through & Pass TYPE IT; Gadelius Medical, Tokyo, Japan, or ADflap; Century Medical, Tokyo, Japan) was placed using a stent-in-stent technique. When RBO was caused by biliary sludge or food impaction, the stent lumen was cleared using a retrieval balloon catheter without additional stent placement. In cases of severe cholangitis, endoscopic nasobiliary drainage (ENBD) was initially performed, followed by definitive stent placement in a subsequent session. The reintervention strategy was determined by the endoscopist based on the biliary anatomy, cause of RBO, initial stent characteristics, and the patient’s clinical condition.

### 2.4. Definitions

Reintervention was defined as the first endoscopic intervention for RBO after initial EUS-HGS, regardless of whether a new stent was placed, and included stent exchange, additional stent placement, balloon cleaning, and ENBD tube placement. Definitive biliary drainage was defined as the placement of a new biliary stent or balloon cleaning alone without additional stent placement, performed during or after the initial reintervention for RBO. Technical success of the initial reintervention was defined as successful completion of biliary drainage through the ESCR during the first reintervention session, including temporary ENBD placement. Technical success of definitive biliary drainage was defined as successful biliary drainage through the ESCR by either stent placement or balloon cleaning alone, including procedures performed during subsequent endoscopic sessions when required. Clinical success was defined as improvement in cholangitis or a ≥50% reduction in serum total bilirubin within 2 weeks. RBO was defined according to the TOKYO Criteria 2024 as stent occlusion or migration requiring biliary drainage or stent removal after technical and clinical success [18]. TRBO was defined as the interval from definitive biliary drainage to the occurrence of RBO [18]. For the Cox regression and Kaplan–Meier analyses of TRBO, patients without RBO were censored at death or the last follow-up. For the competing-risk analysis, death before RBO was treated as a competing event, whereas patients without RBO or death were censored at the last available follow-up. AEs were graded according to the American Society for Gastrointestinal Endoscopy lexicon guidelines [19]. Overall survival (OS) was defined as the interval from definitive biliary drainage to death from any cause. Patients who were alive were censored at the last follow-up.

ESCR formation was assessed by contrast injection during reintervention, and absence of contrast leakage into the peritoneal cavity was considered indicative of successful ESCR formation [1]. This assessment was performed only in the initial PS group after stent removal, because the presence of an indwelling SEMS precluded reliable assessment of contrast leakage. Ascites was assessed using computed tomography before reintervention. Mild ascites was defined as fluid confined to either the upper or lower abdominal involvement, moderate ascites as fluid present in both the upper and lower abdominal involvement, and severe ascites as fluid extending continuously from the pelvic cavity to the upper abdominal involvement [20].

### 2.5. Study Outcomes

Patients were classified into two groups according to the stent type used for initial EUS-HGS: the initial PS group and the initial SEMS group. The primary outcome was the association between the initial stent type and stent patency after definitive biliary drainage, assessed using TRBO and the cumulative incidence of RBO. Secondary outcomes included technical success and procedure-related AEs of the initial reintervention; technical and clinical success, procedure-related and stent-related AEs, incidence of RBO, and OS after definitive biliary drainage; and an exploratory analysis of potential factors associated with TRBO after definitive biliary drainage.

### 2.6. Statistical Analysis

Continuous and categorical variables were compared using the Mann–Whitney U test and Fisher’s exact test, respectively. All tests were two-sided, and a *p*-value < 0.05 was considered statistically significant. TRBO was assessed only in patients who achieved clinical success. TRBO after definitive biliary drainage was evaluated using the Kaplan–Meier method, and survival curves were compared using the log-rank test. The association between the initial stent type and TRBO was evaluated using Cox proportional hazards regression, and hazard ratios (HRs) with 95% confidence intervals (CIs) were calculated. The proportional hazards assumption for the Cox model was assessed visually using log-minus-log survival plots. No missing data were present for the variables included in the analyses. Multivariable Cox proportional hazards regression was performed with adjustment for age (≥65 vs. <65 years), moderate-to-severe ascites (yes vs. no), and duodenal stent placement (yes vs. no). These covariates were selected a priori based on their clinical relevance rather than statistical significance in baseline comparisons. A competing-risk analysis was also performed, with death without RBO treated as a competing event. Cumulative incidence functions were compared using Gray’s test. Fine–Gray subdistribution hazard regression was used to estimate subdistribution hazard ratios (sHRs) and 95% CIs for RBO. The multivariable Fine–Gray model was adjusted for the same prespecified covariates. Post hoc sensitivity analyses were conducted to explore the potential influence of additional confounding factors on the association between initial stent type and subsequent RBO. These analyses considered HGS route (B2 vs. B3), treatment period (July 2016–December 2023 vs. January 2024–January 2026), and primary disease (pancreaticobiliary cancers vs. others). In each post hoc sensitivity analysis, the same prespecified covariates as in the primary analysis were retained, with one additional potential confounding factor included at a time. As an exploratory analysis, univariable Cox proportional hazards regression was performed to assess potential clinical factors associated with TRBO after definitive biliary drainage. Given the limited number of RBO events, no additional multivariable model incorporating these exploratory variables was constructed. OS after definitive biliary drainage was estimated using the Kaplan–Meier method, and survival curves were compared using the log-rank test. All statistical analyses were performed using EZR (version 1.61; Saitama Medical Center, Jichi Medical University, Saitama, Japan) [21].

## 3. Results

### 3.1. Patient Characteristics

Among 170 patients who underwent initial EUS-HGS between July 2016 and January 2026, 69 patients who developed RBO and underwent reintervention through the ESCR were included (Figure 1). Of these, 23 patients had initially received PS and 46 had received SEMS. Among patients who did not develop RBO after EUS-HGS, 26 (100%) in the initial PS group and 74 (98.7%) in the initial SEMS group died without experiencing RBO (*p* = 1.00). The remaining patient in the initial SEMS group was still alive at the time of analysis.

Baseline characteristics at reintervention are shown in Table 1. There were no significant differences in age, sex, ECOG performance status, primary disease, site of biliary obstruction, surgically altered anatomy, ascites, and duodenal stent placement between the groups. B3 access was significantly more common in the initial SEMS group than in the initial PS group (91.3% vs. 52.2%, *p* < 0.01). The median TRBO of the initial stent was 62 days (95% CI, 41–73) in the initial PS group and 88 days (95% CI, 51–105) in the initial SEMS group, with no significant difference between the groups (log-rank *p* = 0.38). This analysis was restricted to patients who developed RBO, rather than the entire cohort of patients who underwent initial EUS-HGS. Stent occlusion was the predominant cause of RBO in both groups. However, the underlying causes of stent occlusion differed between the groups, with debris more frequently observed in the initial PS group and hyperplasia in the initial SEMS group.

### 3.2. Clinical Outcomes of Reintervention

Clinical outcomes of reintervention are summarized in Table 2. The technical success rate of initial reintervention was 97.1% overall and did not significantly differ between the initial PS and SEMS groups (100% vs. 95.7%, *p* = 0.55). Initial reintervention failed in two patients in the initial SEMS group because of unsuccessful ENBD insertion through the stent lumen in one and stent lumen obstruction by debris preventing device insertion in the other. Both patients subsequently underwent successful stent placement during a subsequent endoscopic session. The rate of ESCR formation was 87.0% in the initial PS group. The ESCR was not established in three patients in this group: one underwent reintervention 18 days after initial stent placement without ascites, whereas the other two underwent reintervention more than 2 months after initial stent placement and had severe ascites. ENBD tube placement was more frequent in the initial SEMS group (52.2% vs. 26.1%, *p* = 0.04). Median procedure time did not differ significantly between the groups (25 vs. 29 min, *p* = 0.40). No procedure-related AEs occurred.

### 3.3. Clinical Outcomes of Definitive Biliary Drainage

Clinical outcomes of definitive biliary drainage are shown in Table 3. Among the 46 patients in the initial SEMS group, 15 underwent balloon cleaning alone without placement of a new biliary stent. The median follow-up period after definitive biliary drainage was 108 days overall and did not differ significantly between the groups. Definitive biliary drainage was technically successful in all 69 patients, including the two patients in whom initial reintervention had been unsuccessful. Clinical success rate was 89.9% overall and was comparable between the groups (91.3% vs. 89.1%, *p* = 1.00). Of the seven patients who did not achieve clinical success, five had persistent cholangitis requiring additional percutaneous or transpapillary biliary drainage. One patient had persistent hyperbilirubinemia and received best supportive care (BSC) without further intervention due to poor general condition. The remaining patient experienced stent migration and underwent PS exchange. RBO occurred in 38.1% of patients in the initial PS group and 46.3% in the initial SEMS group (*p* = 0.60). The number of reinterventions after definitive biliary drainage did not differ significantly between the groups. No procedure-related AEs occurred. One patient in the initial SEMS group developed a moderate stent-related AE (liver abscess), which was treated with antibiotics. Following definitive biliary drainage, systemic chemotherapy was initiated or resumed in approximately half of the patients in each group, with no significant difference between the groups (*p* = 1.00).

### 3.4. Overall Survival and Stent Patency After Definitive Biliary Drainage

Median OS after definitive biliary drainage was 83 days (95% CI, 49–247) in the initial PS group and 132 days (95% CI, 83–208) in the initial SEMS group (*p* = 0.38). Among the 62 patients who achieved clinical success after definitive biliary drainage, RBO occurred in 8 of 21 patients in the initial PS group and 19 of 41 patients in the initial SEMS group. Death before RBO occurred in 10 and 19 patients, respectively. Three patients in each group were censored for other reasons. In the initial PS group, one patient underwent surgery and two remained alive without RBO at the end of follow-up. In the initial SEMS group, two patients were lost to follow-up and one underwent surgery. The cumulative incidence of RBO did not differ significantly between the groups when death without RBO was treated as a competing event. The 90-day cumulative incidence of RBO was 24.3% (95% CI, 8.4–44.6%) in the initial PS group and 31.7% (95% CI, 18.1–46.2%) in the initial SEMS group. There was no statistically significant difference in the cumulative incidence of RBO between the groups over the follow-up period (Gray’s test, *p* = 0.62). Initial SEMS placement was not significantly associated with the cumulative incidence of RBO in the adjusted Fine–Gray model (adjusted sHR, 1.67; 95% CI, 0.70–3.97) (Figure 2A). Similarly, Kaplan–Meier analysis showed no significant difference in TRBO between the groups. Median TRBO was 160 days (95% CI, 82–not reached) in the initial PS group and 140 days (95% CI, 59–350) in the initial SEMS group. Initial SEMS placement was not significantly associated with TRBO in the adjusted Cox model (adjusted HR, 1.32; 95% CI, 0.55–3.13) (Figure 2B). Post hoc sensitivity analyses additionally adjusting for HGS route, treatment period, or primary disease did not identify a statistically significant association between initial stent type and subsequent RBO. However, the estimates were imprecise, particularly after adjustment for treatment period (Supplementary Table S1).

In the initial PS group, RBO occurred in 5 of 14 patients who underwent PS exchange and 3 of 7 patients who underwent SEMS exchange. No statistically significant differences in the cumulative incidence of RBO or TRBO were observed between these strategies in the unadjusted analyses (Figure 3A,B). In the initial SEMS group, RBO occurred in 10 of 25 patients who underwent additional PS placement, 2 of 3 patients who underwent additional SEMS placement, and 7 of 13 patients who underwent balloon cleaning alone. No statistically significant differences in the cumulative incidence of RBO or TRBO were detected among these strategies in the unadjusted analyses (Figure 3C,D). These subgroup analyses were exploratory and involved small numbers of patients and RBO events, particularly in the additional SEMS group (*n* = 3). Therefore, although no statistically significant differences were observed, the limited sample size and event counts precluded a reliable assessment of differences in outcomes among reintervention strategies. Among the three patients who underwent additional SEMS placement, one underwent the procedure because of stent migration, resumed chemotherapy thereafter, and subsequently developed RBO at 130 days. The remaining two patients underwent additional SEMS placement because of hyperplasia and subsequently received best supportive care. Of these, one developed RBO after 28 days, whereas the other died without RBO at 49 days.

Exploratory univariable Cox proportional hazards regression analysis was performed to identify clinical factors associated with TRBO after definitive biliary drainage in patients who achieved clinical success. None of the evaluated factors were significantly associated with TRBO (Table 4).

## 4. Discussion

In this study, we compared reintervention outcomes and stent patency after definitive biliary drainage according to the initial stent type (PS or SEMS) used for EUS-HGS. High technical success rates of endoscopic reintervention through the ESCR were observed after both initial PS and SEMS placement. Initial stent type was not significantly associated with RBO after definitive biliary drainage in either Cox proportional hazards or competing-risk analyses. Although the initial stent type may influence the technical approach to reintervention, no statistically significant association between initial stent type and subsequent stent patency was detected. However, the wide confidence intervals indicate that clinically meaningful differences between the groups cannot be excluded.

Reintervention after EUS-HGS differs according to the initial stent type. PS can generally be removed, allowing direct access through the ESCR, whereas reintervention after SEMS placement often requires access through the existing stent lumen or stent mesh [7,8,9]. Complete ESCR formation was observed in 87.0% of patients in the initial PS group. Among the three patients with incomplete ESCR formation, one underwent early reintervention, whereas the other two had severe ascites, suggesting that ESCR formation may be influenced by both the time interval after stent placement and the presence of ascites. Nevertheless, successful reintervention was achieved by carefully advancing a guidewire through the incompletely formed ESCR. Conversely, two reintervention failures occurred after SEMS placement because of difficulty in device passage through the stent, although definitive stent placement was subsequently achieved in both patients. These findings highlight the distinct technical challenges associated with reintervention after initial PS and SEMS placement, despite the high technical success rates observed in both groups. SEMS removability may also be important when repeated interventions are anticipated. A previous study reported that removal and replacement of PCSEMS during reintervention were associated with longer post-reintervention TRBO than interventions without stent removal, whereas tissue hyperplasia was associated with unsuccessful SEMS removal [22]. Therefore, when selecting a SEMS, removability and accessibility for future reintervention should be considered in addition to primary stent patency.

Previous studies have generally demonstrated longer primary stent patency with SEMS than with PS after initial EUS-HGS [23,24]. Another study reported median TRBOs of 183 and 92 days with SEMS and PS, respectively, although stent patency was similar within the first 100 days and AEs were more frequent with SEMS [25]. These studies evaluated outcomes after initial EUS-HGS, whereas the present study specifically examined reintervention outcomes and stent patency after definitive biliary drainage. In the present study, no statistically significant association was detected between initial stent type and subsequent RBO after successful reintervention. However, the limited sample size and wide confidence intervals prevented a definitive assessment of the association between initial stent type and subsequent stent patency. At successful reintervention, subsequent stent patency may be influenced by factors other than the initial stent type, including the reintervention strategy and characteristics of the newly placed stent.

No statistically significant differences in the cumulative incidence of RBO or TRBO after definitive biliary drainage were observed among the different reintervention strategies. However, the subgroup sample sizes were small, particularly for additional SEMS placement (*n* = 3), limiting the interpretation of these exploratory findings. In clinical practice, the reintervention strategy should be tailored to the cause of RBO. Balloon cleaning may be sufficient for sludge-related obstruction or food impaction when the existing SEMS remains functional, whereas stent exchange or additional stent placement may be preferable for tissue hyperplasia, tumor ingrowth, or tumor overgrowth. Similarly, no statistically significant association between the evaluated clinical factors and TRBO was detected in the exploratory analysis. Given the limited number of RBO events, however, these findings should be considered hypothesis-generating. The competing-risk analysis, which accounted for death without RBO, also showed no significant association between initial stent type and the cumulative incidence of RBO.

From a clinical perspective, initial stent selection should balance primary stent patency with the feasibility of future reintervention. As advances in systemic therapy prolong OS, patients with MBO may increasingly require durable biliary drainage and repeated reinterventions during their disease course [16]. In patients with a relatively favorable prognosis who are likely to require repeated biliary interventions, PS may be considered when removability and accessibility for future reintervention are important considerations, as PCSEMS may be difficult to remove. Conversely, in patients with advanced disease and limited life expectancy, SEMS may be considered as an initial option because of their longer primary stent patency. This may be particularly relevant in patients with malignant duodenal obstruction requiring duodenal stenting [26,27]. SEMS may also be considered in patients with ascites along the puncture route, in whom prevention of bile leakage is particularly important [28].

Our study has several limitations. First, it was a retrospective, single-center study with a relatively small sample size, and initial stent selection and reintervention strategy were at the discretion of the treating endoscopist. Differences in the EUS-HGS route (B2/B3), underlying malignancies, and systemic treatments, together with heterogeneous reintervention strategies, may have introduced residual confounding despite adjustment for prespecified variables. Second, the analysis was restricted to patients who developed RBO after initial EUS-HGS and underwent reintervention through the ESCR. Although all patients who developed RBO underwent ESCR reintervention, restricting the analysis to patients with RBO may have introduced selection bias and limits the generalizability of the findings. Third, initial stent type was strongly associated with treatment period, with SEMS predominantly used in the earlier period and PS in the later period. This substantial imbalance and limited overlap between groups raise concerns about confounding by temporal changes in devices, procedural techniques, operator experience, and systemic therapy. Although post hoc sensitivity analyses were performed, they could not fully address this potential confounding. In addition, reintervention strategies differed according to initial stent type, and some strategies were not applicable to both groups. Therefore, the independent effects of initial stent type and reintervention strategy could not be reliably separated. Finally, the limited number of RBO events reduced the precision of adjusted estimates, as reflected by the wide confidence intervals, and limited the statistical power of subgroup and exploratory analyses. Although death without prior RBO was treated as a competing event in the Fine–Gray analysis, the high mortality rate further complicates the interpretation of stent patency outcomes. Larger prospective multicenter studies are warranted to clarify the association between initial stent type and reintervention outcomes.

## 5. Conclusions

High technical success rates of endoscopic reintervention through the ESCR were observed after both initial PS and SEMS placement. Initial stent type was not significantly associated with subsequent RBO after definitive biliary drainage in either Cox proportional hazards or competing-risk analyses. However, the limited sample size, wide confidence intervals, and potential temporal confounding prevented a definitive assessment of the association between initial stent type and subsequent RBO. Further studies are needed to clarify the relationship between initial stent type and subsequent reintervention outcomes.

## Figures and Tables

**Figure 1:**
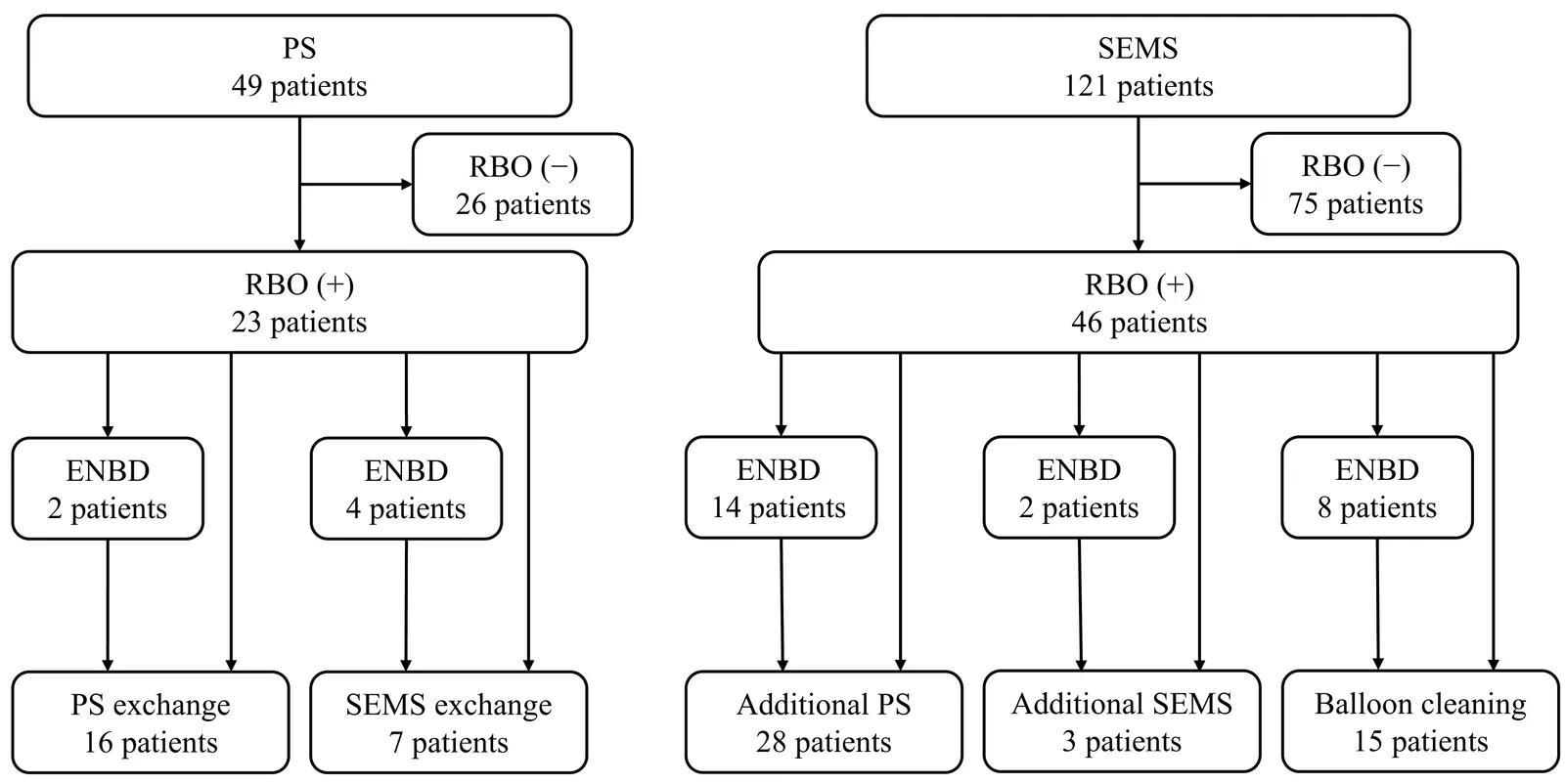
Patient flowchart. ENBD; endoscopic nasobiliary drainage, PS; plastic stent, RBO; recurrent biliary obstruction, SEMS; self-expandable metallic stent.

**Figure 2:**
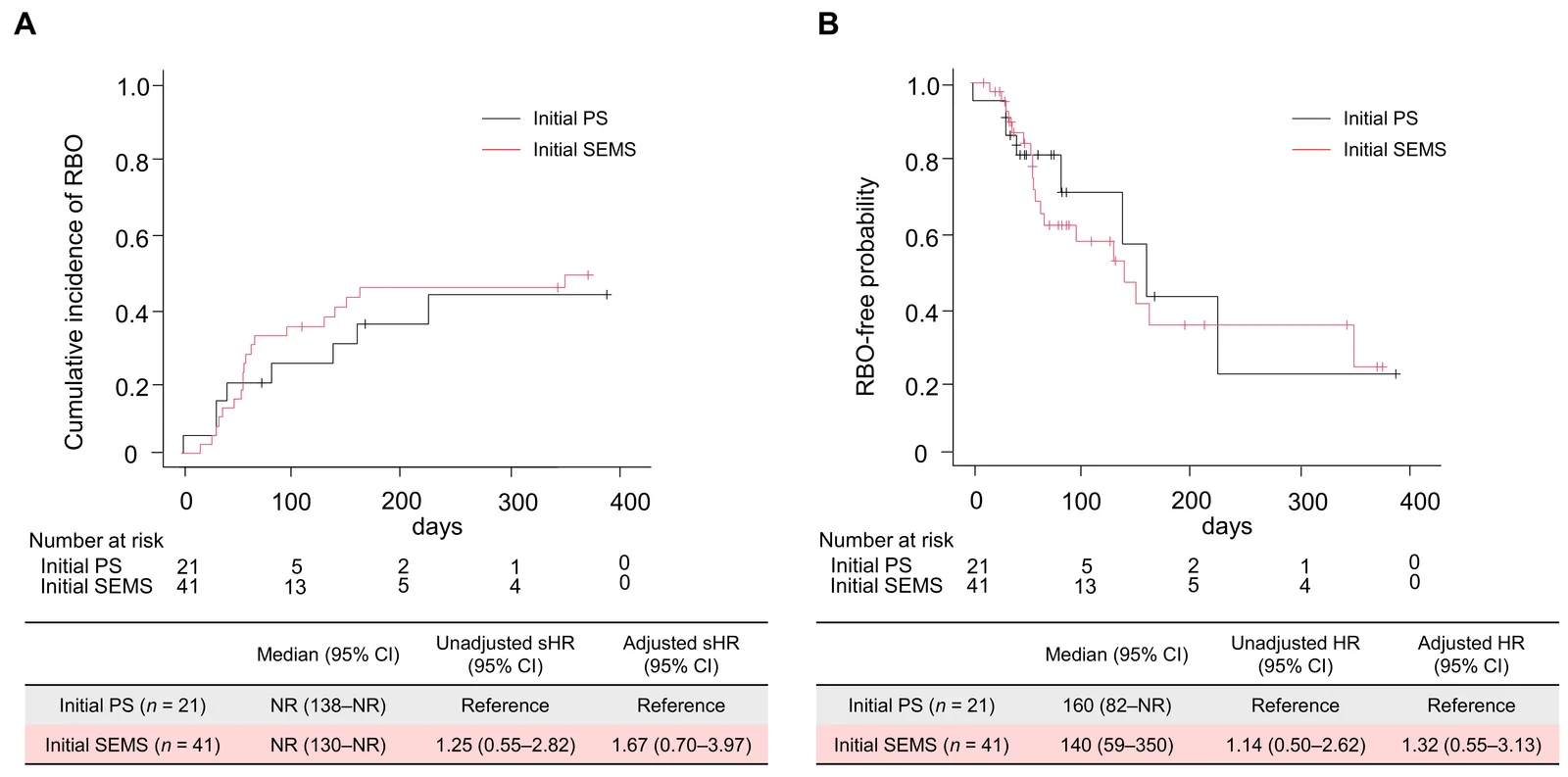
Time to recurrent biliary obstruction after definitive biliary drainage according to the initial stent type. (**A**) Cumulative incidence curves for RBO and (**B**) Kaplan–Meier curves for TRBO. Black and red lines represent the initial PS and SEMS groups, respectively. CI; confidence interval, HR; hazard ratio, NR; not reached, PS; plastic stent, RBO; recurrent biliary obstruction, SEMS; self-expandable metallic stent, sHR; subdistribution hazard ratio, TRBO; time to recurrent biliary obstruction.

**Figure 3:**
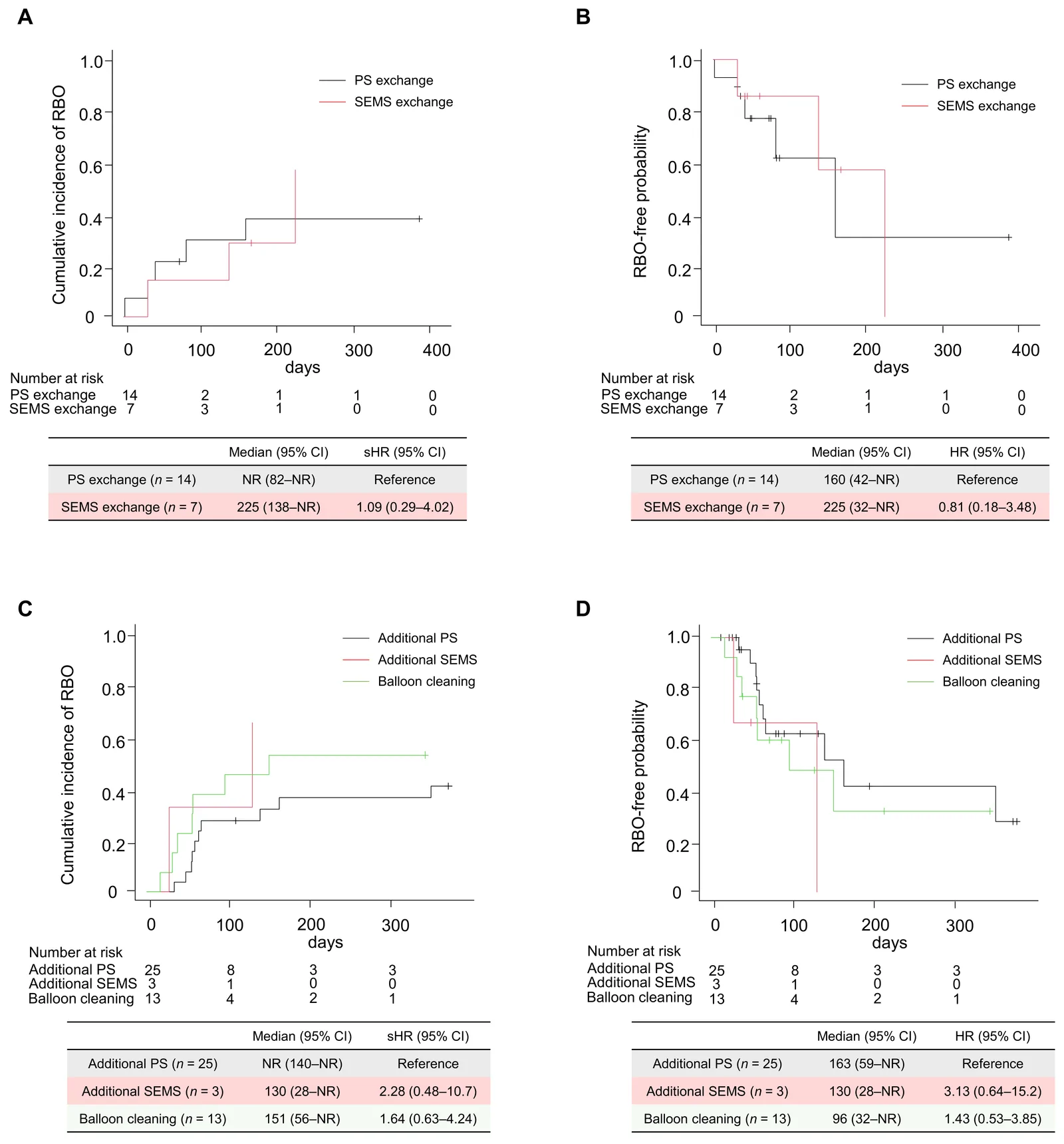
Time to recurrent biliary obstruction according to the reintervention strategy. (**A**) Cumulative incidence curves for RBO and (**B**) Kaplan–Meier curves for TRBO comparing PS exchange and SEMS exchange in the initial PS group. (**C**) Cumulative incidence curves for RBO and (**D**) Kaplan–Meier curves for TRBO comparing additional PS placement, additional SEMS placement, and balloon cleaning in the initial SEMS group. In panels A and B, the black and red lines represent PS exchange and SEMS exchange groups, respectively. In panels C and D, the black, red, and green lines represent additional PS placement, additional SEMS placement, and balloon cleaning groups, respectively. CI; confidence interval, HR; hazard ratio, NR; not reached, PS; plastic stent, RBO; recurrent biliary obstruction, SEMS; self-expandable metallic stent, sHR; subdistribution hazard ratio, TRBO; time to recurrent biliary obstruction.

**Table 1:** Patient characteristics at reintervention.

	Initial PS (*n* = 23)	Initial SEMS (*n* = 46)	*p*-Value
Age	65 (38–81)	66.5 (38–92)	0.97
Sex, male	12 (52.2)	27 (58.7)	0.61
ECOG performance status, 0/1/2/3	9/11/3/0 (39.1/47.8/13.1/0)	11/29/4/2 (23.9/63.0/8.7/4.4)	0.39
Primary disease			0.05
Pancreatic cancer	10 (43.5)	32 (69.5)	
Bile duct cancer	8 (34.8)	11 (23.9)	
Gastrointestinal cancers	4 (17.4)	2 (4.4)	
Hepatocellular carcinoma	0	1 (2.2)	
Cancers of unknown primary	1 (4.3)	0	
Site of biliary obstruction			
Hilar/Distal	10/13 (43.5/56.5)	12/34 (26.1/73.9)	0.17
Surgically altered anatomy, yes	4 (17.4)	5 (10.9)	0.46
Ascites			
None/mild/moderate/severe	9/7/4/3 (39.1/30.4/17.4/13.1)	15/12/9/10 (32.6/26.1/19.6/21.7)	0.77
Moderate-to-severe ascites	7 (30.4)	19 (41.3)	0.43
Duodenal stent, yes	3 (13.0)	11 (23.9)	0.35
HGS route, B2/B3	11/12 (47.8/52.2)	4/42 (8.7/91.3)	<0.01
TRBO of initial stent, days *	62 (95% CI, 41–73)	88 (95% CI, 51–105)	0.38
Causes of RBO of initial stent			0.32
Stent occlusion	20 (87.0)	42 (91.3)	
Debris	20	19	
Tumor ingrowth	0	3	
Hyperplasia	0	20	
Stent migration	2 (8.7)	4 (8.7)	
Other	1 (4.3)	0	

Numbers are shown in *n* (%) or median (range), unless otherwise indicated. * TRBO is presented as the Kaplan–Meier estimate of the median with its 95% CI and was compared using the log-rank test. CI, confidence interval; ECOG; Eastern Cooperative Oncology Group, HGS; hepaticogastrostomy, PS; plastic stent, SEMS; self-expandable metallic stent, TRBO; time to recurrent biliary obstruction.

**Table 2:** Clinical outcomes of reintervention.

	Initial PS (*n* = 23)	Initial SEMS (*n* = 46)	*p*-Value
Technical success	23 (100)	44 (95.7)	0.55
ESCR formation, yes	20 (87.0)		
ENBD tube placement, yes	6 (26.1)	24 (52.2)	0.04
Duration of ENBD tube placement, days	6.5 (4–13)	4.5 (1–50)	0.94
Procedure time, min	25 (10–68)	29 (6–102)	0.40
Procedure time, ≥30 min	8 (34.8)	22 (47.8)	0.44
Procedure-related adverse events	0	0	

Numbers are shown in *n* (%) or median (range). ENBD; endoscopic nasobiliary drainage, ESCR; endosonographically created route, PS; plastic stent, SEMS; self-expandable metallic stent.

**Table 3:** Clinical outcomes of definitive biliary drainage.

	Initial PS (*n* = 23)	Initial SEMS (*n* = 46)	*p*-Value
Type of stent used for definitive biliary drainage			
7Fr 14 cm Through & Pass TYPE IT	15	12	
7Fr 7–12 cm Through & Pass		10	
8.5Fr 7–15 cm Through & Pass	1	3	
7Fr 7, 15 cm Through & Pass Double Pigtail		2	
7Fr 9 cm ADflap		1	
6 mm 10, 12 cm HANAROSTENT Biliary Multi-Hole Benefit	5		
8 mm 10 cm Niti-S Spring Stopper	2		
8 mm 8 cm HANAR OSTENT		1	
8 mm 8 cm Niti-S Large Cell D-type		2	
Balloon cleaning alone	0	15 (32.6)	<0.01
Follow-up period, days	76 (27–388)	129.5 (12–2142)	0.24
Technical success	23 (100)	46 (100)	
Clinical success	21 (91.3)	41 (89.1)	1.00
RBO *	8 (38.1)	19 (46.3)	0.60
Procedure-related adverse events	0	0	
Stent-related adverse events	0	1 (2.2)	1.00
Liver abscess		1	
Number of reinterventions after definitive biliary drainage	0 (0–9)	0 (0–11)	0.70
Systemic chemotherapy after definitive biliary drainage	12 (52.2)	24 (52.2)	1.00

Numbers are shown in *n* (%) or median (range). * RBO was evaluated only in patients who achieved clinical success. PS; plastic stent, RBO; recurrent biliary obstruction, SEMS; self-expandable metallic stent.

**Table 4:** Exploratory univariable analysis of clinical factors associated with time to recurrent biliary obstruction after definitive biliary drainage.

Univariable Analysis		
Risk Factors	HR (95% CI)	*p*-Value
Age, ≥65 years	0.67 (0.31–1.43)	0.30
Sex, male	1.05 (0.48–2.31)	0.89
HGS route, B3	0.70 (0.29–1.68)	0.43
ECOG performance status, 0 or 1	1.36 (0.31–5.79)	0.67
Primary disease, pancreaticobiliary cancers	0.95 (0.28–3.20)	0.94
Site of biliary obstruction, distal	0.59 (0.26–1.29)	0.18
Surgically altered anatomy, yes	1.18 (0.44–3.13)	0.73
Initial stent type, PS	0.87 (0.38–1.99)	0.74
TRBO of initial stent, ≥4 months	0.60 (0.25–1.44)	0.25
Duodenal stent at reintervention, yes	0.78 (0.23–2.65)	0.69
Moderate-to-severe ascites, yes	0.82 (0.30–2.20)	0.70
ENBD tube placement at reintervention, yes	0.79 (0.36–1.75)	0.57

CI; confidence interval, ECOG; Eastern Cooperative Oncology Group, ENBD; endoscopic nasobiliary drainage, HGS; hepaticogastrostomy, HR; hazard ratio, PS; plastic stent, TRBO; time to recurrent biliary obstruction.

## Data Availability

The data presented in this study are available on request from the corresponding author due to privacy and ethical restrictions.

## Supplementary Materials

The following supporting information can be downloaded at https://www.mdpi.com/article/10.3390/jcm15218711/s1. Table S1: Primary and post-hoc sensitivity analyses of the association between initial stent type and recurrent biliary obstruction after definitive biliary drainage.
