# Supplementary material for: Comparison of Reintervention Outcomes After Endoscopic Ultrasound-Guided Hepaticogastrostomy According to the Type of Initial Stent
Source: J Clin Med. 2026 Nov 8;15(21):8711. doi: 10.3390/jcm15218711 (PMC13763880; doi:10.3390/jcm15218711)
Supplement: Supplementary file 1 [file jcm-15-08711-s001.zip › jcm-4602779-supplementary.pdf]

Supplementary Material

Comparison of Reintervention Outcomes After Endoscopic Ultrasound-Guided Hepaticogastrostomy according to the Type of Initial Stent

Table S1. Primary and post-hoc sensitivity analyses of the association between initial stent type and recurrent biliary obstruction after definitive biliary drainage

Analysis	N	RBO events	Adjustment variables (in addition to initial stent type)	Adjusted HR (95% CI)	Adjusted sHR (95% CI)
Primary analysis	62	27	Age ≥65 years; moderate-to-severe ascites; duodenal stent placement	1.32 (0.55–3.13)	1.67 (0.70–3.97)
Sensitivity 1: HGS route	62	27	Age ≥65 years; moderate-to-severe ascites; duodenal stent placement and HGS route	1.86 (0.64–5.40)	2.34 (0.77–7.10)
Sensitivity 2: Treatment period	62	27	Age ≥65 years; moderate-to-severe ascites; duodenal stent placement and treatment period	1.76 (0.23–13.3)	2.59 (0.27–24.1)
Sensitivity 3: Primary disease	62	27	Age ≥65 years; moderate-to-severe ascites; duodenal stent placement and primary disease	1.40 (0.55–3.56)	1.65 (0.60–4.55)

CI, confidence interval; HGS, hepaticogastrostomy; HR, hazard ratio; PS, plastic stent; RBO, recurrent biliary obstruction; SEMS, self-expandable metallic stent; sHR, subdistribution hazard ratio.

The initial PS group was used as the reference category in all analyses. The primary analysis was adjusted for age ≥65 years, moderate-to-severe ascites, and duodenal stent placement. In the post hoc sensitivity analyses, the same adjustment variables were retained, and each analysis additionally included one potential confounding factor: HGS access route (B2 vs. B3), treatment period (July 2016–December 2023 vs. January 2024–January 2026), or primary disease (pancreaticobiliary cancers vs. others). Cox proportional hazards models were used to estimate HRs, and Fine–Gray competing-risk models were used to estimate sHRs, treating death before RBO as a competing event. All sensitivity analyses were exploratory, and the estimates should be interpreted cautiously because of the limited number of events and potential residual confounding. The reference categories for the covariates were age <65 years, no moderate-to-severe ascites, no duodenal stent placement, HGS route B2, the earlier treatment period (July 2016–December 2023), and pancreaticobiliary cancers, respectively.
